# Implementing health promotion in schools: protocol for a realist systematic review of research and experience in the United Kingdom (UK)

**DOI:** 10.1186/2046-4053-1-48

**Published:** 2012-10-20

**Authors:** Mark Pearson, Roy Chilton, Helen B Woods, Katrina Wyatt, Tamsin Ford, Charles Abraham, Rob Anderson

**Affiliations:** 1 PenTAG, Institute of Health Services Research, University of Exeter Medical School, University of Exeter, Exeter, EX2 4SG, UK; 2 School of Health and Related Research, University of Sheffield, Sheffield, S1 4DA, UK; 3 Institute of Health Services Research, University of Exeter Medical School, University of Exeter, Exeter, EX2 4SG, UK; 4 PenTAG, Peninsula College of Medicine and Dentistry (PCMD), Veysey Building, Salmon Pool Lane, Exeter, EX2 4SG, UK

**Keywords:** Health promotion, Schools, Implementation, Realist review, Systematic review

## Abstract

**Background:**

School-based interventions and campaigns are used to promote health and address a wide variety of public health problems. Schools are considered to be key sites for the implementation of health promotion programmes for their potential to reach the whole population in particular age-groups and instil healthy patterns of behavior early in life. However, evidence for the effectiveness of school-based health promotion interventions is highly variable. Systematic reviews of the evidence of school-based interventions tend to be highly problem- or intervention- specific, thereby missing potential generic insights into implementation and effectiveness of such programmes across problems.

**Methods/design:**

A realist systematic review will be undertaken to explain how, why and in what circumstances schools can provide feasible settings for effective health promotion programmes in the United Kingdom (UK). The review will be conducted in two phases. Phase 1 will identify programme theories about implementation (ideas about what enables or inhibits effective health promotion to be delivered in a school setting). Phase 2 will test the programme theories so that they can be challenged, endorsed and/or refined. A Review Advisory Group of education and health professionals will be convened to help identify and choose potential programme theories, provide a ‘reality check’ on the clarity and explanatory strength of the mechanisms to be tested, and help shape the presentation of findings to be usable by practitioners and decision-makers. Review findings will be disseminated through liaison with decision-makers, and voluntary and professional groups in the fields of education and health.

## Background

Schools provide the setting for promoting health and preventing a very wide variety of public health problems, which include obesity, smoking, sexual health, injury prevention, physical activity, diet, mental health, depression and bullying. Whether national or locally driven, the appeal and possible rationales for such initiatives could be:

they are universal, capturing the whole population in the relevant age-group [1]

they provide an opportunity to ‘set’ healthy patterns of behavior early in school children’s development, which may last throughout life [1,2]

they capitalize on school children being a captive audience

schools and schoolchildren can be used as a catalyst for change in families and the wider community [1]

in schools, some sensitive health issues, such as sexual health and contraception, can be handled without parental oversight

teachers or school peers may be more effective at delivering certain types of health message or changing health-related attitudes than others; conversely, outsiders in the classroom may work better

However, evidence for the effectiveness of school-based health promotion interventions is highly variable [3,4]. Also, systematic reviews of the evidence of school-based interventions tend to be highly problem- or intervention- specific, thereby missing generic insights about the implementation and effectiveness of such programmes across problems. We shall conduct a realist systematic review [5], informed by a completed review of previous systematic reviews, to endeavor to explain how, why and in what circumstances schools can provide a feasible setting for effective health promotion programmes in the UK. A realist approach will enable us to locate and synthesize evidence across different fields of health promotion practice, and produce nuanced insights into the implementation on:

1. Key school or wider environmental contexts.

2. Required steps in the implementation process.

3. The nature of health promotion activities which are suited to different types of schools.

A realist systematic review aims to attain a contextualized understanding of how and why complex interventions achieve particular effects (in realist language, how mechanisms lead to outcomes in particular contexts). This understanding is pursued by testing ‘programme theories’, which are often expressed as a model linking outcomes to programme activities and the underlying theoretical assumptions [6]. Contained within programme theories, even if not explicitly stated, are ideas about how a problem can be best addressed and how factors that may undermine the actions of the programme can themselves be addressed [7]. Realist review methods have been specifically advocated for evaluating evidence about complex interventions and their implementation [5,8], and can provide explanatory insight into variations in the effectiveness of programmes that, for example, a Cochrane review of randomized controlled trials may be less likely to provide [9].

It is not appropriate at the outset of a realist review to attempt to specify whether posited factors are mechanisms or contexts, since it is the purpose of the review to develop an understanding of how these factors operate in conjunction with one another. It is this understanding that will enable such factors to be classified as ‘mechanisms’ (how a programme’s resources or opportunities interact with the reasoning of individuals and lead to changes in behavior) or ‘contexts’ (the wider configuration of factors, not necessarily connected to a programme, which may enable or constrain the operation of specific mechanisms). However, some possible factors that could explain how the implementation of health promotion in schools can be facilitated or hindered are listed below. This is a provisional and indicative list, which will be revised in the course of the review:

practicalities of fitting a programme into the timetable of the school day

availability of specialist equipment or materials

beliefs and attitudes of teachers and head teachers about the importance of the health problem

content and change targets of the programme

beliefs, attitudes and perceptions of the people delivering the programme

competencies of the people delivering the programme

mode of delivery of the programme

beliefs, attitudes and other characteristics of the school children’s parents

beliefs and attitudes of school children (at different ages) about the importance of health problems or specific behaviors

development of behavioral skills of the children themselves

compatibility with subjects within the National Curriculum or the goals of Personal, Social, and Health and Education (PSHE) and with other initiatives, such as Schools4life or Social and Emotional Aspects of Learning (SEAL)

There are two related and ongoing systematic reviews led by UK-based researchers: (1) Langford *et al.*[10], a Cochrane systematic review evaluating the effectiveness of the World Health Organization (WHO) Health Promoting School framework; and (2) Bonell *et al.*[11], on the health effects of school environments and school environment-based interventions to improve health.

Our inclusion/exclusion criteria are designed to focus the review on the implementation processes of gaining access to schools and working within schools to promote health. This focus will enable the review to maximize the insights gained by looking across health promotion for different topics conducted in schools, and aims to complement the ongoing Langford *et al*. [10] and Bonell *et al.*[11] systematic reviews. We recognize that the extent to which a focus on the implementation (including feasibility and sustainability) of public health programmes can be a separate focus of systematic reviews is a current area of methodological debate and development [12].

In addition to the specific focus of programme implementation, Phase 2 of the review will be exclusively focused on explaining the success of such initiatives in UK schools. This geographical focus is for two reasons. First, it reflects the policy and practice focus of the research funding body (the National Institute for Health Research (NIHR) School for Public Health Research). Second, and arguably more importantly, it reflects a presumption that many of the issues and mechanisms that determine the implementation of health promotion in schools will be context-specific. They will depend on aspects of school life, the curriculum, socio-economic variations and diversity in communities, which are specific to UK primary and secondary schools.

To inform this realist systematic review, we have already conducted a rapid review of systematic reviews to identify the key dimensions on which school-based health promotion programmes differ and identify some initial programme theories (ideas about how programmes aim to achieve their goals).

This systematic review is registered on the PROSPERO database (registration number: CRD42012002640).

### Review objective

The objective is to use a theory-driven evidence synthesis to identify what influences successful implementation of health promotion in UK schools. The review will have two phases. Phase 1 will identify programme theories about implementation (ideas about what enables or inhibits effective health promotion to be delivered in a school setting) from a range of published and other sources. Phase 2 will test these programme theories, using published and unpublished empirical evidence, through the process of reasoning, as detailed under ‘Data synthesis’.

### Review questions

What are the main factors or mechanisms that are thought to explain the success or failure of the implementation of health promotion in schools?

Is there an association between these factors and mechanisms and the successful implementation of health promotion in schools?

What public health problems and in what circumstances do schools provide a feasible and sustainable setting for effective health promotion in the UK?

## Methods

### Search strategy

There will be a number of search phases for Phase 1 (identification of programme theories about implementation in any Organisation for Economic Co-operation and Development (OECD country)) and Phase 2 (testing of programme theories about implementation in the UK only) of the review.

An initial search will be guided by search terms specifying the type of interventions of interest (for example health promotion, health education and health improvement) and the setting (primary or secondary schools). The search will be designed to locate a broad range of sources, including editorials, opinion pieces and commentaries (primarily for identifying programme theories); as well as comparative effectiveness studies, process evaluations and qualitative research (primarily for testing programme theories). Subsequent bibliographic database searches will be developed to locate sources relating to specific aspects of programme theories not identified in the initial search and to locate studies that will enable selected programme theories to be tested.

The development of search terms will also be informed by the findings of our rapid review of reviews and input from our Review Advisory Group. Scoping and test searches will explore the value and limitations of using country filters. The iterative nature of a theory-driven review means that refinements to the search strategy may be required in response to emerging findings. However, an example of the initial search strategy is included in the search protocol (Additional file 1).

The databases to be searched will include, but not necessarily be limited to: Applied Social Science Index and Abstracts (ASSIA), CINAHL, MEDLINE, PsycINFO, Social Science Citation Index (SSCI), Science Citation Index (SCI), Social Policy and Practice; selected Evidence for Policy and Practice Information and Co-ordinating Centre (EPPI-Centre) databases, for example Current Education and Children's Services Research UK (CERUKplus); British Education Index, Education Resources Information Center (ERIC), Database of Abstracts of Reviews of Effectiveness (DARE), Health Technology Assessment (HTA), the Cochrane Database of Systematic Reviews (all in the Cochrane Library) and the Campbell Collaboration. Ongoing programmes from the UK or unpublished evaluations of health promotion in UK schools will be identified by searching NIHR, Department of Health, Department for Education and other research registers or programme funders, where they can be feasibly searched.

Additional strategies will be used to locate additional sources of evidence. Websites and grey literature sources will also be searched, for example the Department of Health and the Department for Education websites. An indicative list of sources is presented in the search protocol (Additional file 1). The precise combination of strategies will depend upon the nature of our findings. We would expect our search to progressively focus in the order in which the strategies are presented below, but this cannot be stated with certainty:

citations contained in the reference lists of included papers

sources identified through contact with professional networks

‘cited by’ articles search

### Study inclusion criteria (Phase 1)

Sources that provide rich descriptions of the delivery of school-based health promotion for children aged 5 to 16 years in any OECD country. These are likely to include editorials, opinion pieces, commentaries, comparative effectiveness studies, process evaluations, qualitative research, programme manuals and systematic reviews. We will include health promotion programmes and activities that are delivered to school children primarily in a school setting, including those outside of school hours.

Since the aim of Phase 1 is to locate a range of programme theories to test, it is not appropriate to foreclose potentially important perspectives by pre-specifying exclusion criteria.

### Study inclusion criteria (Phase 2)

#### Population

##### Inclusion criteria

Children aged 5 to 16 years attending primary or secondary schools in the UK (both state schools and private schools (so-called ‘public’ schools), regardless of Academy status). Since there is no uniformity in the organization of secondary and further education settings, a programme aimed at children aged 5 to 16 years, but which includes a minority of school children aged 17 or 18 years, would still be included.

##### Exclusion criteria

Non-UK schools, pre-schools (and pre-school-age children), sixth form and further education colleges (because most children will be over 16-years-old and the structure, education culture and organization will be different from secondary schools).

#### Intervention

##### Inclusion criteria

Health promotion programmes that are being, or have been, delivered in UK primary or secondary schools.

##### Exclusion criteria

Community-based health promotion programmes, where the school is one of several local settings for promoting the health of both adults and children. In practice, this means that if the primary starting point for a programme is not a school, it would be excluded.

#### Comparator

Children aged 5 to 16 years attending schools in the UK, where a different health promotion programme is delivered, or no health promotion programme is delivered.

Since the testing of programme theories will involve a range of study designs, including process evaluations and qualitative research, the comparator criteria will only be applied to comparative effectiveness studies.

#### Types of study to be included

Comparative effectiveness studies, where intervention and comparator groups are assigned by the research (randomized and non-randomized controlled trials).

Process evaluations, which aim to document how and why school-based health promotion programmes are successfully implemented or not, or are effective or not. These may be quantitative or qualitative, and alongside a comparative effectiveness study or stand-alone.

If a low number of comparative effectiveness studies are located, we shall consider including (in order of preference) uncontrolled before and after studies, and cohort studies.

#### Outcomes

There is likely to be a considerable breadth of topics and health problems in the health promotion programmes evaluated in the included studies, and therefore pre-specification of all outcomes is not appropriate. However, we shall develop a list of outcomes based on those reported in the included sources. We envisage that these outcomes in children aged 5 to 18 years will include:

standardized measures of physical, emotional or mental health

intermediate measures of health, for example observed or self-reported health behaviors or body mass index

health-related knowledge and attitudes

### Quality assessment (Phase 1)

We shall use a hybrid appraisal tool based on previous critical appraisal work [12,13], which enables sources to be classified as a conceptually-rich (thick) or thin (weaker) description. We found this tool to be practical and useful in a recent theory-driven review [14] for the way in which it enabled a focus on the stronger sources of programme theories without simply excluding weaker sources that may still make an important, if lesser, contribution.

### Quality assessment (Phase 2)

For assessing the internal validity of comparative effectiveness studies we propose to use a standard tool for assessing the risk of bias (threats to internal validity). We aim to use the most up-to-date version of the Cochrane risk of bias tool, or an adaptation of it suitable for both randomized and non-randomized studies.

Other study types, such as process evaluations, will be assessed using a modified version of the Wallace *et al.* quality appraisal tool for qualitative studies [15]. This tool encompasses key components of rigor (for example sampling, data collection and data analysis) that are relevant across different fields of research practice. Importantly, given that the number of included studies may be large, the Wallace *et al.* tool focuses on key elements of critical appraisal without becoming so detailed as to become unusable in the context of conducting a review to a reasonable timescale. This tool will also enable us to pinpoint whether or not certain aspects of a study are of higher or lower quality. This is particularly important in a realist review where both relevance and rigor are considered in tandem, meaning that an otherwise poorly conducted study may contribute to the synthesis if the aspect concerned is of sufficient rigor.

### Data extraction (Phase 1)

Consistent with a theory-driven approach, sources that contribute to theory development will be ‘engaged with’ by the reviewers (through a process of note-taking, annotation and conceptualization) rather than ‘extracted’. At the same time we will refine our conceptualization of the key stages, factors or processes which constitute health programme implementation in UK schools.

### Data extraction (Phase 2)

At the stage of testing the programme theories, relevant data/information from included studies and reports will be extracted to a standard data extraction form. A sample of these will be checked by the other reviewer for accuracy, for those data extraction fields which involve quantitative data or key information, such as study design. The data extraction process itself will involve critical discussion between reviewers and the wider team so that data are not simply ‘classified’ but are used to begin to develop a line of argument that feeds into the final synthesis stage.

### Data synthesis

For synthesis, a similar strategy will be used for both Phase 1 (identification of programme theories about implementation) and Phase 2 (testing of programme theories about implementation). Synthesis of the diverse sources of evidence included in a realist review is conducted through a process of reasoning that is structured around the following activities [16]:

juxtaposition of sources of evidence, for example when evidence about implementation in one source enables insights into evidence about outcomes in another source

reconciling of sources of evidence, when results differ in apparently similar circumstances, further investigation is appropriate in order to find explanations for why these different results occurred

adjudication of sources of evidence, on the basis of methodological strengths or weaknesses

consolidation of sources of evidence, when evidence about mechanisms and outcomes is complementary and enables a multi-faceted explanation to be built

situating sources of evidence, when outcomes differ in particular contexts, an explanation can be constructed of how and why these outcomes occur differently

The transparency of a synthesis in a realist review is achieved by documenting these reasoning processes, describing how they are grounded in the empirical evidence and the justification of inferential shifts that occur through this engagement with the evidence.

In addition for Phase 2, we aim to code studies according to: (1) the presence/absence of selected theoretical mechanisms and contexts that are believed to promote successful/unsuccessful programme implementation in schools, and (2) markers of successful implementation. For example, the proportion of schools approached who accepted the intervention and the proportion of schools that initially accepted the intervention, but went on to withdraw or not deliver it fully in some way. As a possible final phase of evidence synthesis, these combinations of mechanisms, contexts and markers of implementation success may be used to try and explain between study (or between programme) differences in programme effectiveness. We hope that this might be achieved through tabulating these data and findings, graphically exploring potential associations or possibly by more formal techniques, such as Qualitative Comparative Analysis [17,18].

### Review advisory group

As well as being based on documentary sources and their synthesis, as described in this protocol, the selection and prioritization of programme theories will be informed by two or three meetings with a Review Advisory Group. It is intended that at least three members of the Review Advisory Group will be directly from schools or the education sector (for example local education authority department, and/or head teachers, PSHEleaders or special educational needs co-ordinators). The Review Advisory Group will help identify and choose amongst different potential programme theories, and provide a ‘reality check’ on the clarity and explanatory strength of the selected theories/mechanisms which become a focus of Phase 2 of the review.

Separately from the Review Advisory Group, we shall also involve and share emerging findings with some school children, through School Councils or recruited through a local ‘family faculty’ (which has been established for the purposes of public involvement in other research conducted by Peninsula Medical School, Universities of Exeter and Plymouth).

### Dissemination

Provision of a review ‘evidence summary’ and liaison with voluntary and professional groups in the fields of:

1. Primary and secondary education, for example British Educational Research Association (BERA) and National Association for Special Educational Needs (NASEN).

2. Behavior change, for example UK Society for Behavioural Medicine (UKSBM) and The British Psychological Society (BPS).

3. Public health, for example The Royal Society for Public Health (RSPH).

4. Mental health, for example Association of Child and Adolescent Mental Health (ACAMH), Royal College of Psychiatrists (RCPsych) and YoungMinds.

These lists will be expanded as part of the work of the Review Advisory Group. Other key decision-makers who contribute to the policy making process (for example in the Department of Health and Department for Education) will be identified so that dialogue can be initiated and taken forward with regard to the review’s findings.

## Abbreviations

ASSIA: Applied Social Sciences Index and Abstracts; CERUKplus: Current Education and Children's Services Research UK; DARE: Database of Abstracts of Reviews of Effectiveness; EPPI-Centre: Evidence for Policy and Practice Information and Co-ordinating Centre; ERIC: Educational Resources Information Center; HTA: Health Technology Assessment; NIHR: National Institute for Health Research; OECD: Organisation for Economic Co-operation and Development; PSHE: Personal Social, Health education; SCI: Science Citation Index; SEAL: Social and Emotional Aspects of Learning; SSCI: Social Science Citation Index; WHO: World Health Organization.

## Competing interests

The authors declare that they have no competing interests.

## Authors' contributions

MP led the design and drafting of the review protocol. RC contributed to the design and drafting of the review protocol. HBW scoped and designed the search strategy. KW, TF and CA provided substantive topic-specific input that informed the revision and refinement of the review protocol. RA conceived the review, oversaw the development and revision of the protocol, and provided substantive methodological input. All authors read and approved the final manuscript.

## Supplementary Material

Additional file 1 Search Protocol: Implementing health promotion in schools: a realist systematic review of research and experience in the UK. Click here for file
