# Supplementary material for: Implementing health promotion in schools: protocol for a realist systematic review of research and experience in the United Kingdom (UK)
Source: Syst Rev. 2012 Oct 20;1:48. doi: 10.1186/2046-4053-1-48 (PMC3488465; doi:10.1186/2046-4053-1-48)
Supplement: Additional file 1 — Search Protocol: Implementing health promotion in schools: a realist systematic review of research and experience in the UK. [file 2046-4053-1-48-S1.pdf]

Search Protocol: Implementing health promotion in schools: a realist systematic review of research and experience in the UK

The proposed review questions are:

a) What are the main factors or mechanisms that are thought to explain the success or failure of the implementation of health promotion in schools?
b) Is there an association between these factors and mechanisms and the successful implementation of health promotion in schools?
c) For what public health problems and in what circumstances do schools provide a feasible and sustainable setting for effective health promotion in the UK?

This document proposes a possible search methodology for both the components of this review. Following agreement of this document, the contents will be placed in the study protocol. Final searches cannot necessarily be determined at this stage as the concepts and relevance need to be explored throughout the search process; therefore any additional search strategies will be agreed within the team and in discussion with the Review Advisory Group as the project progresses.

Proposal for Systematic Review searches

An initial search strategy will be developed in order to retrieve papers for both Phase 1 – identification of programme theories about implementation and Phase 2 – testing of programme theories about implementation. The initial search strategy of free text and subject headings will be developed using terms identified from papers included in the Review of Reviews and from the search strategies employed in the ongoing systematic review of health promotion in schools (Bonell et all, 2011). In order to retrieve a diverse range of evidence to inform both parts of the review no study filters will be applied. The searches will be limited to English language only where possible. This limit will be applied as the review aims to generate an understanding of the implementation of health promotion interventions in schools in contemporary UK settings. A broad range of sources will be searched to reflect the multidisciplinary nature of the review; covering health, social science and educational evidence.

It is anticipated that additional searching may be required to fully explore the evidence base. Further iterations of searching would be developed by an examination of the evidence that emerges from the initial search leading to the generation of additional search strategies; for example to search for further evidence on specific programme theories identified in the initial search. It is not possible to specify these search strategies a priori.

Combination of terms initial search strategy

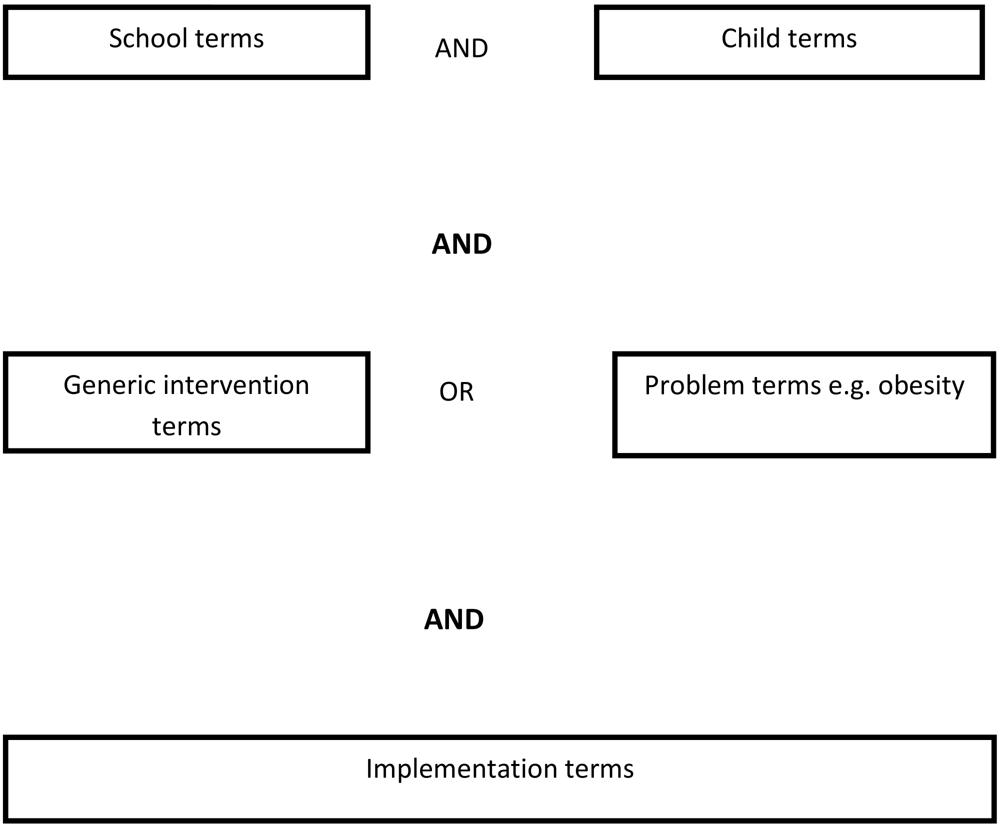

Additional Search techniques

Additional techniques to identify evidence to inform the review will be used. Which techniques and the order in which they will be employed cannot be stated with certainty at this stage in the process.

Additional techniques may include:

- Identification of evidence through contact with professional networks
- Citation searching of included papers in Web of Knowledge and/or Scopus
- Searching reference lists of relevant systematic reviews retrieved through database searches
- Searching reference lists of included papers
- Searching for programmes/interventions identified through database searches
- Searching for key authors
- Searching using index and free text terms of key papers identified by initial search
- Hand-searching of key journals

Grey Literature and Web Searching

In addition to bibliographic database searches web searching will be undertaken to identify evidence not indexed in bibliographic databases, such as ongoing programmes from the UK, or unpublished evaluations of health promotion in UK schools. Websites and grey literature sources include but are not limited to:

- Department of Health: http://www.dh.gov.uk
- Department for Education: http://www.education.gov.uk/
- NIHR Public Health Research Programme: http://www.phr.ac.uk/
- Public Health Observatories: http://www.apho.org.uk/
- NHS Evidence http://www.evidence.nhs.uk
- evaluations of specific programmes, e.g. 'health schools plus'
- Scottish Government: http://home.scotland.gov.uk/
- Welsh Assembly Government: http://wales.gov.uk/?lang=en
- Public Health Wales: http://www.wales.nhs.uk/sitesplus/888/home
- Public health agency: http://www.publichealth.hscni.net/
- The Campbell Collaboration: http://www.campbellcollaboration.org/

Commercial search engines such as "Google" or "Bing" will only be used to identify specific topic relevant websites which will be examined and searched within if further web searching is required. This will be decided within the project team.

Sample search strategy for Medline – Initial search

Database: Ovid MEDLINE(R) In-Process & Other Non-Indexed Citations and Ovid MEDLINE(R) <1946 to Present>

Search Strategy:

----

1 (child* or young adult or young people or pupil* or student* or adolescen* or teenage* or girl* or boy* or youth*).ti,ab.

2 Child/

3 Adolescent/

4 1 or 2 or 3

5 (school* or junior high or teacher* or teaching assistant or educational setting*).ti,ab.

6 exp Schools/

7 5 or 6

8 4 and 7

9 (health promotion or illness prevention or health inequalit* or intervention or multi-component or multi-modal or life skills or social skills or social competence or social influence or social-cognitive or refusal skills or resistance skills or resistance training or shame reduction or harm* minimi?ation or harm* reduction or anger management or anger control or empathy training or cognitive training or affective imagery training or role rehearsal or psycho-educational or behavio?r change or peer-led or teacher-led or partnership-based or internet based or psycho-social or multi-component or self-control).ti,ab.

10 Health Promotion/

11 (mental health or wellbeing or self-esteem or self-confidence or assertiveness or risk taking or victimi?ation or obesity or weight loss or overweight or waist circumference or BMI or healthy eating or nutrition or diet or school meal* or school dinner* or physical* activ* or exercise or fitness or physical education or sedentary or smok* or tobacco or cigarette* or substance misuse or substance abuse or street drug or illegal drug* or illicit drug* or cannabis or marijuana or inhalant* or alcohol or binge drink* or bully* or bullied or bullies or violen* or aggress* or delinquen* or classroom behavio?r or accident prevention or injury prevention or dog bite* or contracepti* or pregnan* or sexual activity or sexually transmitted disease* or sexually transmitted infection or dating violence or risk taking or sexual abuse).ti,ab.

12 *Mental health/ or *Obesity/pc or *weight loss/ or *Overweight/pc or *body mass index/ or *exp exercise/ or *exp Sports/ or *physical fitness/ or exp *Smoking/pc or *Substance-Related Disorders/pc or *exp street drugs/ or *Alcohol Drinking/pc or juvenile delinquency/ or *Violence/pc

or *Accident Prevention/ or *Contraception/ or *Pregnancy in Adolescence/pc or *Sexual Behavior/ or *Sexually Transmitted Diseases/pc or *child abuse/pc

13 9 or 10 or 11 or 12

14 (implementation or feasibility or sustainability or acceptability).ti,ab.

15 8 and 13 and 14

16 limit 15 to english language

Table One – Databases to be searched

Medline and Medline in Process via Ovid
CINAHL via EBSCO
British Nursing Index via Ovid
British Education Index via ProQuest
Cochrane Library, including DARE, CENTRAL, HTA and CDSR via Wiley
ERIC via ProQuest
Australian education index via ProQuest
ASSIA via Proquest
Sociological Abstracts via Proquest
Social Policy and Practice via Ovid
PsycINFO via Ovid
HMIC via Ovid
Science Citation Index via Web of Knowledge (Thomson ISI)
Social Science Citation Index via Web of Knowledge (Thomson ISI)
EPPI Centre Databases – Bibliomap, DoPHER, TRoPHI, The database on Obesity and Sedentary behaviour studies, Schemes to promote healthy weight among obese and overweight children in England, Database of Education Research, Current Educational Research in the United Kingdom (CERUK)<br>http://eppi.ioe.ac.uk/

Table Two – Search terms to inform initial search strategy

Health Promotion generic intervention terms	"Problem" terms	School and Children terms	"Real World" Terms: Implementation and Sustainability
health promotion	mental health	Child	Implementation
illness prevention	wellbeing	Young adult	external validity
health inequality	self-esteem	Young people	generalizability
intervention	self-confidence	Pupil	transferability
multi-component	assertiveness	Student	acceptability
multi-modal	risk taking	Adolescent	practical
life skills	victimisation	Teenage	sustainability
social skills	obesity	Girl	quality control
social competence	weight loss	Boy	embedded
social influence	overweight	youth	
social-cognitive	waist circumference	School	
refusal skills	BMI	Junior high	
resistance skills	healthy eating	Teacher	
resistance training	nutrition	Teaching	
shame reduction	diet	assistant	
harm minimisation	school meal	Educational	
harm reduction	school dinner	setting	
anger management	physical activity		
anger control	exercise		
empathy training	fitness		
cognitive training	physical education		
affective imagery training	sedentary		
role rehearsal	smoking		
psycho-educational	tobacco		
behaviour change	cigarette		
peer-led	substance misuse		
teacher-led	substance abuse		
partnership-based	street drug		
internet based	illegal drug		
psycho-social	illicit drug		
multi-component	cannabis		
self-control	marijuana		
	inhalant		
	alcohol		
	binge drink		
	bullying		
	violence		
	aggression		
	delinquency		
	classroom behaviour		
	accident prevention injury		
	prevention		
	dog bite		
	contraception		
	pregnancy		
	sexual activity		
	sexually transmitted disease sexually		
	transmitted infection dating violence		
	risk taking		
	sexual abuse		

References

Bonell C, Harden A, Wells H, Jamal F, Fletcher A, Petticrew M, Thomas J, Whitehead M, Campbell R, Murphy S, Moore L. Protocol for a systematic review of the effects of schools and school-environment interventions on health: evidence mapping and syntheses. BMC Public Health 2011; 11: 453

Langford R, Campbell R, Magnus D, Bonell CP, Murphy SM, Waters E, Komro KA, Gibbs LF. The WHO Health Promoting School framework for improving the health and well-being of students and staff. Cochrane Database of Systematic Reviews 2011, Issue 1. Art. No.: CD008958. DOI: 10.1002/14651858.CD008958
